# Implementing Information and Communication Technology to Support Community Aged Care Service Integration: Lessons from an Australian Aged Care Provider

**DOI:** 10.5334/ijic.2437

**Published:** 2017-04-10

**Authors:** Heather E Douglas, Andrew Georgiou, Amina Tariq, Mirela Prgomet, Andrew Warland, Pauline Armour, Johanna I Westbrook

**Affiliations:** 1 School of Psychology and Exercise Science, Murdoch University, AU; 2 Centre for Health Systems and Safety Research, Macquarie University, AU; 3 School of Public Health and Social Work, Queensland University of Technology, AU; 4 Uniting, Sydney, AU

**Keywords:** Information and Communication Technology, Home care services, Integrated Care, Information Exchange, Case Study

## Abstract

**Introduction:**

There is limited evidence of the benefits of information and communication technology (ICT) to support integrated aged care services.

**Objectives:**

We undertook a case study to describe carelink+, a centralised client service management ICT system implemented by a large aged and community care service provider, Uniting. We sought to explicate the care-related information exchange processes associated with carelink+ and identify lessons for organisations attempting to use ICT to support service integration.

**Methods:**

Our case study included seventeen interviews and eleven observation sessions with a purposive sample of staff within the organisation. Inductive analysis was used to develop a model of ICT-supported information exchange.

**Results:**

Management staff described the integrated care model designed to underpin carelink+. Frontline staff described complex information exchange processes supporting coordination of client services. Mismatches between the data quality and the functions carelink+ was designed to support necessitated the evolution of new work processes associated with the system.

**Conclusions:**

There is value in explicitly modelling the work processes that emerge as a consequence of ICT. Continuous evaluation of the match between ICT and work processes will help aged care organisations to achieve higher levels of ICT maturity that support their efforts to provide integrated care to clients.

## Introduction

Population ageing is a significant issue facing Australia and countries worldwide [1]. Community aged care services are particularly valuable to help people with functional restrictions who wish to stay in their own homes [2]. Community aged care services are provided by a multidisciplinary team of healthcare professionals, dispersed across large geographical areas, requiring frequent and synchronous communication with other members of staff to effectively provide care to clients [3,4,5]. From July 2015, the Australian community aged care sector additionally faces new challenges associated with the introduction of a Consumer Directed Care model [6]. Under this model, aged care clients have access to greater choice and control over the types of care they receive, how and by whom it is delivered. Despite the assumed benefits to the consumer, aged care providers in Australia have expressed concern about their ability to flexibly address the needs of clients under a Consumer Directed Care model [7]. Providers have also voiced concerns over the potential impact of Consumer Directed Care models on service provision infrastructure and sustainability [7]. In this challenging service provision and policy context, aged care organisations need ways to enhance the delivery of efficient services that meet their clients’ needs.

### What is integrated care and why is it important to community aged care?

Consumer Directed Care models require aged care providers to be more responsive to clients’ expressed needs, to provide individualised care planning, and to maintain a more flexible service delivery infrastructure [7]. Such requirements present aged care providers with the opportunity of instituting integrated models of care. Integrated care models are designed to address issues of continuity of care, efficiency, and effectiveness of services. Kodner and Spreeuwenberg [8] define integrated care as a coherent set of methods and models on multiple levels, to create connectivity, alignment, and collaboration between care providers, in order to improve outcomes for clients and other service users. In their Rainbow Model of Integrated Care, Valentjin et al. [9] define integrated care as a network of multiple professionals and organisations across the health and social care system that provide accessible and comprehensive services to a population in the community. Achieving integration in community aged care appears to have important benefits for elderly individuals living in the community. Integrated care programs for frail elderly populations have demonstrated an impact on the number and duration of short-term hospitalisations, drug use, mortality, cost of services, and a smaller proportion of older people wishing to be institutionalised [10].

Information and communication technology systems can underpin effective integration of care. The Rainbow Model of Integrated Care suggests that *functional integration* is a vital component of integrated care. Functional integration has been defined as the key support activities structured around the primary process of service delivery, to coordinate decision-making between professionals within a care system [11]. Information and communication technology is one method by which functional integration can be achieved [12]. Beland and Hollander [13] reviewed integrated care models for the frail elderly, and identified information and communication technology as one of the key components in achieving such care models. Some of the systems they identified were designed to track clinical information (i.e., electronic health records, standardised computerised clinical charts), while others were introduced to serve administrative functions (i.e., client assessment, classification, cost and functional status databases). For service organisations, information and communication technology can serve as a powerful tool to manage information about clients. This information can also be used to identify client needs and deliver services in co-ordinated and efficient ways [14,15]. Despite international evidence of the potential benefits of information and communication technology systems as an enabler of increased access, greater efficiency, and more effective information flow [16,17], the community aged care sector has been a slow adopter of information and communication technology. There is a corresponding lack of information in the aged care literature about how to achieve functional integration using information and communication technology.

### How information and communication technology might support integration of community aged care services

Information and communication technology systems can support more efficient information exchange [18,19]. In a qualitative study conducted within seven Australian residential aged care facilities, Georgiou et al. [19] found that the information exchange process was typically arranged around the coordination of care, and could occur through multiple channels depending on the type of information that is being communicated. When this information exchange process works well, care is usually effectively and efficiently provided. When this information is exchanged poorly, the quality of care can be reduced [20,21]. Evidence from other industries using information and communication technology to support information exchange suggests that the benefits can take time to emerge and might be the result of many rounds of evaluation [22]. This is consistent with the view that functional integration using information and communication technology will develop over time [23]. Community aged care services hoping to derive benefits associated with information exchange will need to carefully plan the implementation of information and communication technology and institute multiple rounds of evaluation to address problems that emerge over time.

Evidence about how to design, deploy, and maintain information and communication technology applications in community aged care is sparse [24]. The integrated care literature does not address in detail the process of introducing information and communication technology, particularly how information and communication technology changes work processes. Aged care organisations in Australia are increasingly discussing the importance of information and communication technology [25]. Much of the research effort to date has been invested in examining these technologies in residential aged care facilities [26]. Internationally, research on information and communication technology applied to gerontology is dominated by telemedicine and home monitoring techniques for the elderly, in contrast to solutions that enhance care planning, communication and information sharing among professional staff working in community aged care settings [27,28]. Barriers to successful information and communication technology implementation include a failure to account for the interdisciplinary nature of aged care, the community environment in which it is based, and the difficulty this introduces in exchanging information between multiple care providers within the organisation providing services to the same client [29]. A US report on the implementation of information and communication technology in long-term care settings concluded that there was limited understanding of the workflow processes on the frontline of care that might impact on information and communication technology design and implementation in this sector [30]. Exploring the perspectives of key individuals using information and communication technology within the organisation is a vital component of developing technology solutions that support information exchange in health care organisations.

We undertook a case study to describe carelink+, a centralised client service management system implemented by Uniting, a large community aged care service provider. We sought to explicate the care-related information exchange processes associated with carelink+, to identify lessons for organisations attempting to use information and communication technology as a functional integration tool to support community aged care work and service integration for clients.

## Method

### Introduction to the case

In response to the Australian Government “Living Longer, Living Better” legislation that came into effect in August of 2013 [31], Uniting centralised their operational model to support standardising the client journey through community care services. The objective of this model was twofold: 1) to increase the visibility of client information to achieve more effective person-centred care that empowered their clients to make choices about their own wellbeing and health; and 2) to create a sustainable service delivery model that allowed staff to adapt to changing service environments and consistently deliver services efficiently, effectively, and safely. Under this new integrated care model, Uniting introduced a centralised service centre with a single point of contact for all stakeholders, standardised the work processes of care staff in different regions to support the centralised service centre, shifted the administrative and rostering functions to the centralised service centre, and made significant investments in technology infrastructure to support this centralisation. Carelink+ was introduced as the system to enable this standardised and centralised operating model. Carelink+ was designed by IconGlobal as a generic community care service management system [32].

**Figure 1:**
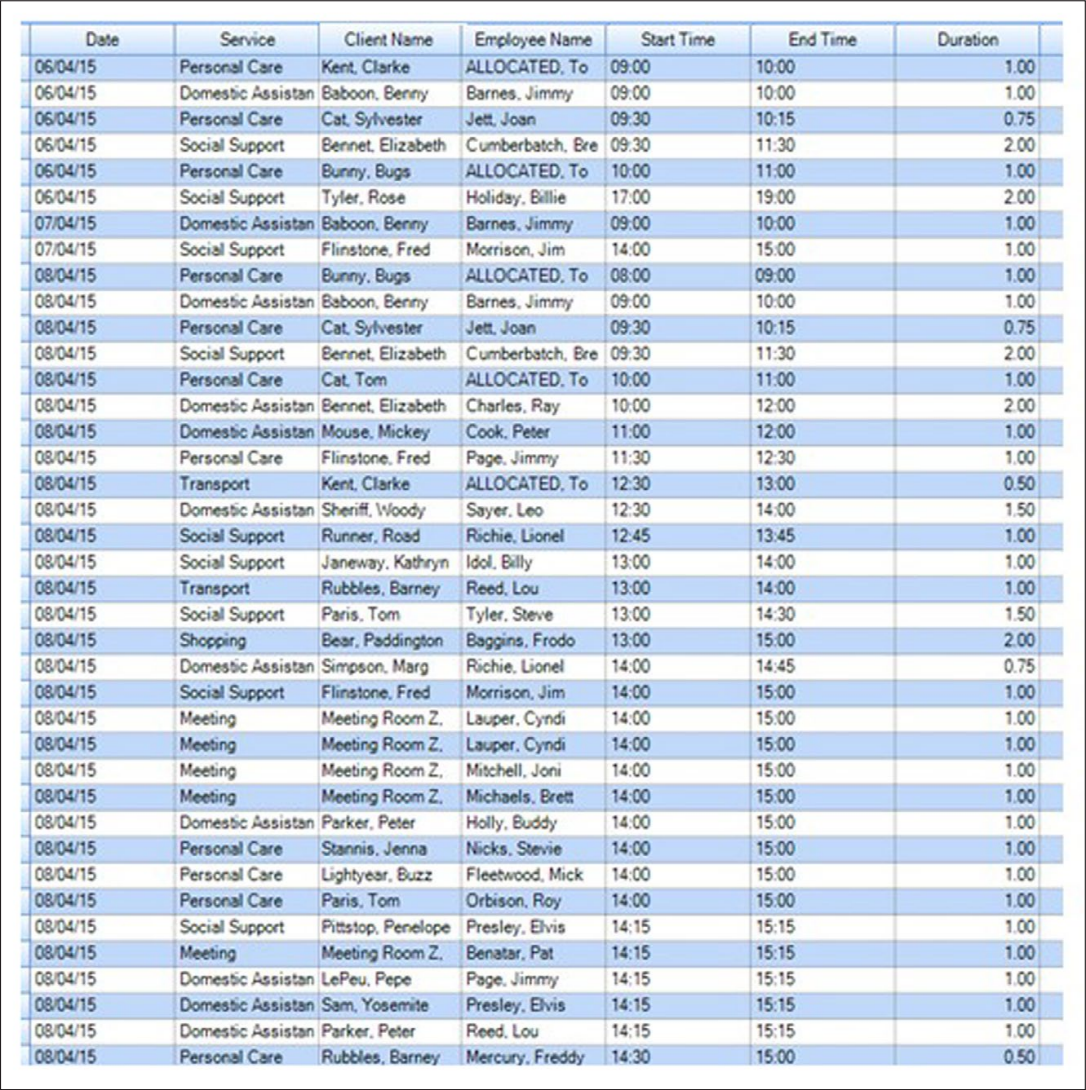
A snapshot of a mock carelink+ roster. **Note:** Funding body has been removed to maintain individual service centre anonymity. Client and employee names were fictional in the carelink+ staff training environment.

During 2012–2013, Uniting provided community aged care services to 7,114 clients. Prior to the introduction of carelink+, client information records were paper-based, and the regional Uniting service centres maintained separate documentation systems for all client records. Carelink+ was designed as a case management and scheduling system to support a single client record. It facilitates the timetabling of services and contains information about both the clients receiving services and the Care Workers scheduled to provide services. Care Workers could either be internal Uniting staff, or external staff employed from outside organisations. Figure 1 gives an example of the service roster information contained in carelink+.

The centralised service centre and carelink+ were introduced by Uniting, with service centre staff who use carelink+ entering information about new clients, rostering services for clients, and responding to care staff queries about client services. Care Workers and Case Managers were expected to use carelink+ to search for care plans, record when services had been delivered, and record the results of client assessments. A proof of concept test for carelink+, including the centralised service centre, commenced in two regional test sites on 19^th^ November and 3^rd^ December 2012. The purpose of carelink+ was to support electronic record keeping and information sharing about clients across the organisation, including Care Workers who provide support services to clients, and Case Managers who coordinate the support services that clients need at the point of care delivery. Carelink+ was also introduced to enable better risk management with visibility over client activity. It achieved this by housing all client data in a centralised system that all staff could access immediately. Before carelink+, Uniting used paper-based forms. Sometimes these forms were located in the client’s home, sometimes they were at a central office, and sometimes they might be located in the back of a Care Worker or Case Managers’ car. This was particularly difficult when multiple members of staff wished to view the files. The roll-out of carelink+ across the organisation was handed over to business as usual operations on 20^th^ March 2015, and it is now a part of the new integrated care model across the three Uniting operating regions in NSW and the ACT.

### Design

We used a case study approach [33] where carelink+ was explored from the perspective of different individuals in the organisation. Purposive sampling was undertaken to ensure that the participant base reflected different levels of involvement with carelink+ across the organisation [34]. We targeted the following individuals: (a) Customer Service Officers (n = 4) who operate the service centre and use carelink+ as the primary information and communication technology system; (b) the Service Manager (n = 1) at the centralised service centre, responsible for the training, development, and support of Customer Service Officers using carelink+; (c) the Information Architect (n = 1) who worked to build a holistic view of Uniting’s work processes, information, and information and communication technology assets; (d) the Implementation Analysts (n = 2), who ensured that deployments of new information and communication technology was carried out correctly, and that existing information and communication technology infrastructure was maintained; and, (e) the Program Manager for Continuous Service Improvement (n = 1), who was responsible for successful delivery of the new integrated care model at Uniting. The views of the staff providing services to clients on the changes to the service model, including Case Workers and Care Managers, are primarily reported elsewhere [35].

### Data collection and analysis

The study comprised seventeen interviews and eleven observation sessions over January – November 2014 carried out by three members of the research team. The interview participants included one Program Manager for Continuous Service Improvement (one interview), one Information Architect (five interviews), two Implementation Analysts (four interviews), the Service Manager (three interviews), and four Customer Service Officers who used the system on a daily basis (four interviews). Multiple interviews were conducted with the same person to capture the changes in work processes over time that were associated with carelink+. Observation sessions were conducted immediately after the interviews when participants provided verbal consent for this to occur. Each professional described and demonstrated their tasks related to introducing carelink+ to support the new integrated care model at Uniting (one session) maintaining the structure underlying carelink+ (four sessions), navigating the carelink+ user interface (three sessions), and explaining a sample of data extracted from the carelink+ database (three sessions). Extensive field notes from the observation sessions were collated immediately after each observation session.

We used a general inductive approach to derive a model of the information exchange processes associated with carelink+ [36]. This approach was used as no model for the use of information and communication technology in the context of community aged care currently exists. Two members of the research team undertook independent close reading of the interview transcripts and observation session field notes, and met to discuss key work processes (themes) described in the data. The information exchange processes that were associated with the introduction of carelink+ from the perspectives of the Customer Service Officers were explicitly modelled based on the analyses. Any differences in the nature of the information exchange processes modelled were discussed during team meetings. The preliminary findings on each aspect of the exploration were relayed back to a committee of three experts within Uniting over six additional meetings spanning twelve months. These three experts were senior members of staff involved in the Community Aged Care and Clinical Governance divisions of Uniting. This provided the opportunity for validation of the findings. This study was approved by the Macquarie University Human Research Ethics Committee.

## Results

Each professional group at Uniting described and demonstrated their tasks related to carelink+. The Program Manager for Continuous Service Improvement described the redesign of Uniting work processes to align with an integrated care model, and the implementation of carelink+ and the centralised service centre to support this new work model. The information and communication technology staff (including the Information Architect and Implementation Analysts) described Uniting activities in terms of the connections between information systems (carelink+ and other information and communication technology systems), and ensuring that the information and communication technology assets aligned with the new work processes described by the community care systems program plan. The Service Manager at the centralised service centre pointed out that understanding the way frontline staff actually work, including the knowledge they had gained from working with carelink+ directly, could be explicitly modelled so that the information was not lost when staff leave the organisation.

“…lack of available information and knowledge [about working with carelink+]. It’s all in people’s heads…there’s been a lot of change in staff as well. So when we do start making traction and getting information, [it] gets lost or not transposed.”

The Service Manager suggested that the implicit information possessed by frontline staff about how carelink+ works and the processes they had developed to use it would be more valuable to the organisation if it was explicitly described and integrated into organisational policy and training documents.

Carelink+ is accessed by all community care staff, from Care Workers, Case Managers, and Customer Service Officers, to the Community Care Director. Care Workers enter information about the services they provided to clients. Case Managers enter information about client assessments and updates to care plans. Customer Service Officers use the information entered by Care Workers and Case Managers to manage changes to client care service rosters. All groups of professionals could identify the roles and responsibilities of other groups. This indicates the existence of inter-professional partnerships in the pursuit of quality for care to aged care clients. The Customer Service Officers were identified as the central point of contact for all staff using carelink+, and their role is described by the following quote.

“…what my role involves is dealing with external stakeholders, and internal stakeholders of the organisation. We have got care workers, clients, case managers, service managers, agency staff as well. So we basically liaise with all these people. And it is basically to provide services to our clients.”

**Figure 2:**
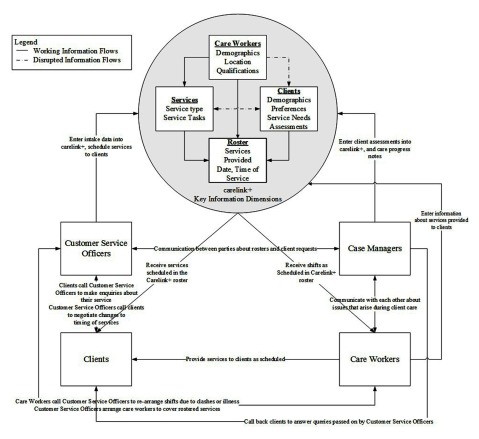
A model of the information exchange process through the key information dimensions of carelink+, frontline staff (Customer Service Officers, Case Managers, and Care Workers), and their relationships.

We modelled the information exchange processes associated with carelink+ from the perspective of the Customer Service Officers. The model derived from the data can be found in Figure 2.

### The model of information exchange at UnitingCare NSW.ACT

The service provision roster is a high volume activity supported by carelink+, as shown in the carelink+ key information dimensions section of Figure 2. The main dimensions that support the roster are: information about the preferences of clients receiving services (Clients), the services available from Uniting (Services), and the preferences of Care Workers who provide services (Care Workers). An algorithm within the software combines these dimensions to produce the service roster by considering the service needs of the client, and which Uniting employees are qualified to provide the services. One Customer Service Officer described the client journey through care and the nature of their role in this journey.

“…so the process is like this. We get a referral from ACAT [Aged Care Assessment Team]…depending on availability, [the client] will be allocated to a service outlet…the Service Managers will decide whether to take on board this client. And once we take on board those clients, initial assessment [in carelink+] over the phone will be done by the Intake Team over here [the centralised service centre]. Once that is all recorded in the system the Case Managers…will go to see the clients personally, and have a more thorough assessment of the client…they will get all the information from the clients as to when they need the service, for how long…they send it through [to] us [through] carelink+, telling us, this client needs service on so and so date. It needs to be with this type of care worker for this – preferably at this time. And we need to try and match that. And then the team over here will create the roster, try and get the appropriate care workers and other care done.”

The quality of entered data has an impact on how effectively each information dimension (Clients, Services, and Care Workers) within carelink+ links together to produce appropriate service provision rosters.

### Barriers to information exchange

Figure 2 also demonstrates the information exchange flows between Customer Service Officers and clients, Case Managers, and Care Workers. Customer Service Officers were the primary contact for all parties about the service provision roster. Clients called them to raise issues with their service arrangements, for example if a Care Worker had not arrived for a planned service. Care Workers called them if they were unable to work, or if they arrived to deliver a service and the client was not home. Case Managers called Customer Service Officers to ask questions about the roster for particular clients, or to request changes to the Care Worker assigned to a client. Conversely, Customer Service Officers called clients to renegotiate service times if a Care Worker was unable to cover their scheduled service. They called Care Workers about providing additional services that were not already allocated to a Care Worker. Finally, Customer Service Officers notified Case Managers when clients requested to speak with them, when a Care Worker could not be found to cover a service, or when a Care Worker was having trouble providing a scheduled service.

The centralised service centre had introduced an unexpected information exchange barrier between clients and care staff. In carelink+, the introduction of the Customer Service Officers meant that clients no longer contacted their Case Managers directly. Instead, they called a Customer Service Officer, who passed on their message to the Case Manager, who then returned the call. Previously, Case Managers coordinated services provided by Care Workers in their region. Clients and Care Workers were also able to call their Case Managers directly. The Program Manager for Continuous Service Improvement indicated that removing the synchronous communication between Case Managers and both Care Workers and clients was an unexpected consequence of the proof of concept test for carelink+. This was rectified following the proof of concept test and pre-implementation communication channels were restored.

“…not all team members were clear on who to contact, resulting in unnecessary call contacts to consumer care direct [the customer service centre]. Case Managers and Care Workers at the proof of concept sites had stopped directly communicating with one another, because they had been told that the centralised service centre would manage their communication instead. After the unmanageable volume of calls to the service centre became apparent, care staff at these sites had to be instructed to resume contacting each other directly.”

#### Data quality

Two examples of a disruption in information exchange were described by Customer Service Officers. The first disruption arose when Customer Service Officers attempted to roster services, depicted by a dashed arrow in Figure 2 between Care Worker and Client information dimensions. Carelink+ is updated with information about the type of Care Worker clients would prefer (for example, male or female), which links with the roster so that Customer Service Officers can select an appropriate Care Worker. This represents an interaction between Clients, Care Workers, and the roster section of the carelink+ Key Information Dimensions shown in Figure 2. This functionality was initially not working in carelink+, meaning that Customer Service Officers had to manually search the available carers list. One of the Customer Service Officers described issues with the quality of the data that prevented this functionality from operating.

“…that feature is not working at this stage. It has got issues and apparently the developers of the program say …the data in carelink+ is not clean. And that is why it is not able to generate that list.”

The Program Manager for Continuous Service Improvement identified that the Customer Service Officers had stopped using this function because data quality was initially problematic. The community care operations project team subsequently engaged in a data quality improvement initiative. The team had to encourage Customer Service Officers to return to this function once the data quality issue had been addressed.

“…now that they’ve had those few experiences of it not working properly they’re a little less excited until it’s proven…”

#### File formats inhibit the visibility of client data

The second disruption was related to the format of files, which impacted the ability of Uniting staff to check that services matched clients’ assessed needs, as shown in Figure 2 by a dashed arrow between Services and Clients. Before prospective clients are referred to a service provider in Australia, they undergo an ACAT assessment with the Aged Care Assessment Team. This assessment contains a record of the problems clients need addressed by community aged care services. It constitutes the basis from which Customer Service Officers perform their initial phone assessment and complete the Intake Priority Form, which supports them to assign a score and a service waitlist position to prospective clients. Both the ACAT assessment and the Intake Priority Form were scanned and then attached to carelink+ in a portable document format (pdf). This meant that the information was inaccessible in a system readable format, which introduced problems with accessing it in carelink+. It also led to additional work for the Customer Service Officers due to a restriction in the size of scanned documents that could be attached to client files.

“…the ACAT assessment that we download from the Medicare website, it’s a 10 page [form], and I can’t really attach it to carelink+, because carelink+ wouldn’t allow me more than two megabytes, and that is about 10 [megabytes]. So what I need to do every time, is I need to print it out, scan it and…compress it, and then save it on my computer and then attach it to carelink+.”

In this case the old operating model of faxing or electronically scanning paper-based documents between Medicare, the Australian government department responsible for aged care funding claims, and the aged care service providers was not compatible with the new IT-enabled operational model of Uniting. Uniting did not rectify this issue upon identifying it because the introduction of the new industry-wide aged care portal MyAgedCare, due for implementation between July and December 2015 [37], would remove the need to attach scanned ACAT assessments to carelink+ from the Medicare website.

The technical issues described above, both to be eventually resolved, had consequences for the workflow of Customer Service Officers, making their tasks more difficult to complete. Such issues are difficult to identify without explicitly accessing the knowledge that key users have about the system. All staff at Uniting acknowledged the value of information exchange at the frontline of community care. The Service Manager at the centralised service centre additionally pointed out that some of the information exchange occurring at the frontline of care was not explicitly described in Uniting workflow descriptions or official training documents as dictated by their community care systems program plan.

## Discussion

This case study described how barriers to information exchange processes were identified during the implementation of large scale information and communication technology and their implications for the workflows of frontline staff. The key findings of this study are summarised in Table 1, and are likely to be relevant to many aged care provider organisations grappling with the role of information and communication technology in achieving integrated models of care. The findings of the current study indicate that evaluation beyond the implementation of information and communication technology itself is necessary to identify unexpected barriers between information and communication technology design and work processes.

**Table 1:** Key Lessons for Information and Communication Technology-Enabled Integration of Care.


1.	As information and communication technology is embedded in business as usual operations, transforming implicit experience gained by frontline users into explicit knowledge will have a significant impact on the efficiency with which new users are trained to use the resource. Explicitly modelling the information exchange processes occurring between frontline staff following the introduction of such technology is particularly important to determine barriers to efficiency.
2.	The introduction of information and communication technology to support service delivery can have unexpected effects that continue beyond the implementation phase. Constant evaluation of information and communication technology as it is integrated into normal business operations is important to identify these effects.
3.	The benefits of information and communication technology will take time and multiple rounds of evaluation to emerge. Aged care organisations who continually evaluate the information and communication technology, and respond to the lessons learned, can achieve more efficient information exchange. This should lead to more effective integrated care for aged care clients.


As has been demonstrated [38] work as performed is often quite different from work as imagined [39,40]. There are large benefits to organisations who transform the implicit knowledge of frontline information and communication technology users into explicit knowledge that is integrated into service provision practices and organisational policy [41]. This further corresponds with the research on enterprise architecture. Enterprise architecture has been defined as “the organising logic for an organisation’s information and communication technology infrastructure and business processes” [42] (p. 98). The intent of enterprise architecture is to identify the key technology, data, and system components that must be shared across multiple parts of the organisation [42]. Enterprise architecture can be considered akin to the functional integration component of the Rainbow Model of Integrated Care using information and communication technology. Carelink+ represents enterprise architecture within Uniting. The organisation identified a need to integrate the information on client demographic data, care plans, and rostering to increase the availability of information across geographically dispersed professionals. The availability of this information from carelink+ has subsequently enhanced synchronous information exchange at Uniting.

The benefits of information and communication technology can take time to emerge. In case studies with eleven organisations working in a variety of industries, Ross and Westerman [43] identified that some of these organisations reported an initial struggle before the benefits of the new information and communication technology solution became apparent. While this outsourcing is not the same as Uniting outsourcing the development of their community care management system to a technology vendor, the key finding remains the same. Prior to the introduction of carelink+ across the organisation, service centres had their own processes for dealing with their client information records, which they maintained themselves. This presented complications for Uniting introducing carelink+ and the associated centralised service centre. Ross and Westerman further found that within each studied organisation, although they experienced difficulties adjusting to an information and communication technology outsourcing model initially, all eleven had experienced benefits by the end of the study. Uniting hopes to achieve benefits to the efficiency and effectiveness of their service provision by using carelink+ as a centralised knowledge repository about their clients. Uniting immediately realised the benefits of increased visibility associated with their client record, and are now working to improve the data quality within carelink+ to support increased efficiency and effectiveness in their service provision. As is the situation with Uniting, moving from an environment of departmental siloes, in which a different technological structure is present in each department, to more standardised technologies and data requirements, needed organisational learning, which takes time [43]. Careful research and an understanding of their business before implementing information and communication technology enabled Uniting to achieve immediate benefits. Further benefits will likely evolve over time.

An example of standardised information and communication technology processes improving integrated care for frail elderly clients comes from the PRISMA project [10], in which a computerised clinical chart was used to ensure all practitioners had quick access, could continuously update information, and could inform other clinicians of a client’s progress and service provision changes using the chart. Uniting had introduced a similar data collection form using carelink+. Even though this standardised form was introduced, individual work groups were still using local rules to enter data [44], which manifested in non-standardised data entry and had subsequent impacts on the ability of Customer Service Officers to deliver services as expected to clients. Before implementation of carelink+, each geographical region of the organisation was autonomous, and they had different standards and systems for entering client data. Uniting are currently working to address these data entry inconsistencies through a continuous quality improvement initiative.

Managing patients’ trajectories is a cooperative exercise [45]. This was the case for Customer Service Officers, who reported being in contact with clients, Care Workers, and Case Managers to update the information in carelink+ and coordinate work schedules. In contrast to clinical perspectives on healthcare work, information and communication technology perspectives view work as discrete tasks for individuals that can be represented as pre-fixed workflows [22]. By imposing this pre-fixed workflow structure, additional burdens on healthcare personnel have been observed in hospitals, home care for frail elderly adults, and the care of patients with stroke [38]. Being responsive to the feedback of frontline staff should result in improvements to the information and communication technology assets that better match the work processes in each setting. This case study described a situation where Customer Service Officers were the central point of contact for Clients, Case Managers, and Care Workers. A limitation in carelink+ associated with file attachments created additional work for Customer Service Officers and did not allow access to the information in the file at a future date. Uniting have identified and addressed these limitations to the extent they are capable. These findings highlight the importance of designing information and communication technology solutions that support work as it is actually performed by frontline staff, and the value to service provider organisations of explicating these work processes. In this case explicating the barrier to accessing information in carelink+ allowed Uniting to directly address the issue with selecting appropriate Care Workers to provide services to clients.

This case study has also demonstrated that considering the needs and requirements of frontline staff using information and communication technology reveals important information that influences the ability of information and communication technology to support integrated models of care. Customer Service Officers needed the ability to automatically search carelink+ for Care Workers that matched client preferences. By identifying that the automatic search function was not working due to inconsistencies in data entry, Uniting could improve data quality so that this function was available to frontline staff. Although carelink+ was not originally developed through end user consultative approaches, it has been designed to be flexible to the identified needs of Uniting. If required, Uniting have the ability to develop customised data collection forms, and at the time of the research study, the developers of carelink+ were incorporating the feedback of Uniting based on user tests into future iterations of the software. Information exchange is a key component of care coordination [46] and subsequent safety and quality of care [47]. This is particularly relevant to the community aged care setting, in which Case Managers and Care Workers are geographically separated, independent, and highly mobile due to the need to provide support services in clients’ homes [3,4]. Inspecting the information exchange processes around information and communication technology systems provides valuable information about possible barriers that have the potential to disrupt workflow and hinder the provision of quality care services to aged clients. The consistency of data entry into the system supported the operation of carelink+ as a functional integration tool at Uniting.

Internationally, aged care organisations are under increasing pressure to respond to a demanding and changing aged care landscape in flexible ways. Enterprise architecture and enterprise architecture maturity, which is defined by Bradley et al. [42] as the set of plans that guide healthcare management responsibilities and strategies, can support such flexibility. Increasing levels of enterprise architecture maturity impacted the effectiveness of 164 US hospitals’ information technology resources in achieving strategic goals [42]. Leveraging such information technology resources by evolving enterprise architecture maturity has the potential to provide organisations in the community aged care sector with the capability to respond and adapt to an increasingly competitive environment [48,49,50]. One of the key stages of enterprise architecture maturity development is the ability to standardise technology to integrate systems and enhance data sharing. Consistent with this stage in enterprise architecture maturity development, carelink+ has allowed Uniting to capitalise on the information they collect on their clients. By collecting information in one place that can be accessed by all relevant staff, increased communication was achieved between Care Workers, Case Managers, and Customer Service Officers. Data sharing and integrity issues were easily identified and resolved, for example standardised data entry across geographic regions of the organisation. The increased visibility of data and the information exchange occurring around it allowed Uniting to increase their information technology efficiency [42]. These gains in efficiency will allow Uniting to enhance the provision of services to clients.

Information and communication technology more generally has the potential to integrate client records and render them fully accessible to multiple users in different geographic areas. These functions support organisational agility by improving communication and the tracking of service provision [51]. Community aged care services are information sensitive activities that exchange vast amounts of data across a large array of services, health care providers, and institutions [52]. The Organisation for Economic and Cooperation and Development has identified the availability and exchange of quality data as a major challenge faced by the sector [53]. This case study has addressed how service provider organisations can adjust their operational model so that the maximum benefit from information and communication technology implementation can be realised. A close examination of the changes to work processes that are influenced by the introduction of information and communication technology will likely assist organisations to address the barriers to providing efficient and effective care, and therefore develop increasing levels of enterprise architecture maturity to support such functions.

Published literature describing how aged care organisations adjust their operational models to capitalise on the benefits of information and communication technology to support the information exchange processes at the frontline of community aged care is sparse and, to our knowledge, the account we present in this paper is one of the first to address this subject. By exploring the experience of Uniting, we identified key lessons about information and communication technology and its support of integrated care while it was being implemented in a community aged care service provider organisation. The results emphasise the role of the frontline users in informing the design of an effective information and communication technology system. Implementation of information and communication technology in community aged care settings more generally should consider the context, particularly the workflow of frontline care staff. The data quality issues preventing Customer Service Officers from automatically searching the database for appropriate Care Workers would not have come to light without asking them directly. It is unlikely that merely implementing an information and communication technology system as purchased will be enough to achieve the expected cost effectiveness and care efficiency benefits [54,55]. Enterprise architecture maturity approaches state that such effectiveness and efficiency benefits should not be expected until alignment between work processes and information and communication technology systems has been achieved. Any benefits from information and communication technology will take time to emerge, after multiple rounds of evaluation. Ultimately, continuing to develop the capability of information and communication technology will result in aged care organisations that have achieved functional integration, and are able to provide all the advantages of integrated care to older people living at home under their care.

Only one organisation was studied in this research, which limits the generalisability of our findings to other community aged care providers. The individuals chosen to participate in this study were primarily from the information technology side of the operational model implementation at Uniting, however other information and communication technology developed for community aged care settings might have a different group of key personnel with different relationships and needs. Further, two of the authors were from Uniting, introducing the potential for conflicts of interest in the reporting of results. These conflicts are unlikely to have affected the results, given that the external research team was responsible for all data collection, analysis, and reporting of results. Despite the limitations, the findings reported in this case study provide valuable insights into the ways in which information and communication technology shapes information exchange processes. These insights can assist in informing future efforts in supporting integrated, quality, and safe care for aged care clients.

## Conclusion

A major obstacle to the uptake of information and communication technology in community aged care has been a limited understanding of the unique and complex needs of the sector, in particular the workflows and processes that might influence information and communication technology design and success in this sector [30]. We have contributed evidence to this area by describing the information exchange processes that were designed to support a centralised community care management system. Investigating the system during its implementation in the organisation highlighted the importance of understanding and incorporating the perspectives of multiple groups. Despite the careful planning that was invested into a new integrated care model at Uniting, there remained barriers to information exchange that were only identified after the system had been implemented. Future research on carelink+ in community aged care could consider if clients or other frontline care providers have been impacted by the introduction of information and communication technology. The key lessons for other service provider organisations attempting to implement functional integration with information and communication technology are that the benefits take time and multiple rounds of evaluation to emerge, and are unlikely to emerge without considering and explicitly modelling the work knowledge and experiences of frontline staff providing care. Information and communication technology appears to support functional integration by enhancing information exchange and collaboration between professionals providing care.
